# Machine learning-assisted prognosis prediction and surgical decision-making for glioblastoma: perceived benefits and concerns of patients, caregivers, and neurosurgeons

**DOI:** 10.3389/fneur.2026.1818670

**Published:** 2026-07-02

**Authors:** Meredith V. Parsons, Olivia Buckley, Hamasa Ebadi, Eric Leuthardt, Tristan McIntosh

**Affiliations:** 1 Bioethics Research Center, Department of Medicine, Washington University School of Medicine in St. Louis, St. Louis, MO, United States; 2 Taylor Family Department of Neurosurgery, Department of Medicine, Washington University School of Medicine in St. Louis, St. Louis, MO, United States

**Keywords:** benefits, clinical decision-making, glioblastoma, machine learning, prognostication, risks, stakeholder engagement

## Abstract

**Introduction:**

It is becoming more common for machine learning (ML) models to aid prognostication and clinical decision-making, including for glioblastoma (GBM) cases. However, there is a lack of empirical research on how end-users view potential benefits and risks of implementing such models in clinical practice.

**Methods:**

This study examines the perspectives of GBM patients (*n* = 13), their caregivers (*n* = 14), and neurosurgeons treating GBM (*n* = 15) about an ML model designed to predict GBM patient prognosis and inform surgical decisions. Participants completed interviews that were audio-recorded, professionally transcribed, and coded by the study team.

**Results:**

All three groups thought a major benefit of the ML model was its ability to take into account a large amount and scope of patient data, which could help facilitate communication and decision-making among patients and neurosurgeons when planning treatment strategies or end-of-life care. Participants also expressed concerns about potential inaccuracies or biases in model output, and shared unease about the possibility of the model replacing a neurosurgeon’s clinical judgment entirely. Some patients and caregivers expressed concern about the model being in early stages of development and about how the delivery of ML-informed prognostic information could cause patients to lose hope or become confused about important prognostic or surgical information.

**Discussion:**

This study highlights the value of engaging multiple stakeholders and triangulating their perspectives when developing ML models to support clinical decision-making. While ML models show great promise in synthesizing large amounts of data and supporting decision-making, biases in the data used to train these models and over-reliance on their predictions risk negatively affecting patient outcomes.

## 1 Introduction

Glioblastoma (GBM) represents the most aggressive primary brain tumor in adults, with a median survival of only 9 to16 months despite maximal treatment (1, 2). The disease exhibits significant heterogeneity in patient outcomes, making accurate prognostic prediction critical for treatment planning and shared decision-making (3). Traditional prognostic methods, including Tumor Node and Metastasis (TNM) staging, Karnofsky Performance Status (KPS), MGMT promoter methylation status, and neurosurgeon clinical experience, provide valuable prognostic information. However, these methods have limited ability to capture biological heterogeneity and insufficient precision for individual GBM patient predictions (4, 5). This creates substantial uncertainty for patients and caregivers, complicating critical treatment decisions and end-of-life planning.

Machine learning (ML) models have emerged as promising tools to address the limitations of traditional prognostic methods. These models can integrate multimodal data and enable greater precision for individual patient predictions, demonstrating greater predictive performance with C-index values of 0.78–0.88 compared to approximately 0.70 for conventional approaches (6, 7). In GBM specifically, deep learning models applied to preoperative MRI have achieved greater than 83% accuracy for survival category classification, while multimodal systems combining radiomics, genomics, and clinical data show potential for risk stratification and personalized treatment strategies (8–10). Realizing this potential for precision medicine and revolutionizing neuro-oncology decision-making depends on understanding how key stakeholders perceive ML-assisted prognosis and its ability to address existing prognostic limitations. Appropriate and responsible clinical integration requires that ML augments, rather than replaces, clinical judgment.

However, the use of ML in prognostication of GBM raises ethical complexities that extend beyond technical accuracy; GBM is a context in which documented challenges of integrating ML in healthcare are compounded by the high stakes and urgency of such a serious terminal illness. In the context of terminal illness, prognostic discussions involve deeply personal, emotional, and value-laden decisions. An ML model that predicts poor prognosis based on maximizing predictive accuracy may provide the “best” information, yet patients may prefer hope and uncertainty over precise survival predictions, exercising their autonomy to choose what information they receive. Similarly, inaccurate ML predictions could harm patients, but failing to use ML when it offers greater accuracy could also cause harm, raising difficult questions about which risk is worse. ML models may prioritize survival duration over quality of life, fail to consider individual patient preferences, or introduce automation bias, which is the tendency to favor suggestions from automated systems over contradictory information from non-automated sources, even when the automated system is incorrect (11, 12).

Evidence from oncology reveals complex dynamics around trust in ML-assisted clinical decision-making. Research shows that patient trust in ML is closely intertwined with trust in their physicians: patients who perceive their clinicians as over-relying on ML without integrating nuanced clinical judgment may experience diminished trust in both the technology and their healthcare providers (12, 13). This dual-trust dynamic is particularly salient in palliative and end-of-life care contexts, where concerns have been raised about ML depersonalizing care and undermining clinician-patient relationships (14, 15). Additionally, algorithmic bias in model development and variable patient comfort with ML present further challenges to establishing trust in these technologies (16, 17). Further, individual patients and caregivers may vary in their trust and comfort with ML-generated prognostic information, influenced by personal beliefs and values.

Multi-stakeholder input is essential for responsible implementation of ML-assisted prognosis. Patients, as the ultimate recipients of prognostic information, must trust and accept these tools for effective shared decision-making. Caregivers serve as key partners in treatment decisions and end-of-life planning. Neurosurgeons play a central role in GBM care and serve as clinical gatekeepers who determine whether and how ML predictions are integrated into clinical workflows. Understanding neurosurgeon perspectives can help address the risk of clinicians either over-relying on ML predictions or dismissing them, anticipate implementation challenges before they arise, and support responsible commercialization.

Despite the critical need for stakeholder input, no studies have explored the perspectives of patients, caregivers, and neurosurgeons on ML-based survival and functional outcome models specifically in the context of GBM. Examining these three perspectives together, rather than in isolation, can demonstrate where different stakeholders align or differ in their risk–benefit assessments, support mutual understanding of varying concerns and benefits, guide development of group-specific educational materials and support resources, and inform strategies as ML prognosis enters routine practice (18). This comprehensive, multi-perspective approach is essential for de-risking implementation and understanding the full ecosystem of trust, acceptance, and integration challenges (19). Thus, we interviewed patients with GBM (*n* = 13), their caregivers (*n* = 14), and neurosurgeons (*n* = 15) in this exploratory qualitative study to understand the perceived benefits and concerns of using a real-world ML survival and functional outcome model currently under development by the Washington University School of Medicine (WUSM) Department of Neurosurgery to predict GBM prognosis and inform surgical decision-making.

## 2 Materials and methods

### 2.1 Participant recruitment

From February to October 2025, the study team recruited GBM patients (*n* = 13), their caregivers (*n* = 14), and neurosurgeons treating GBM patients (*n* = 15) in the US. This study employed purposive homogeneous sampling to prioritize participants with GBM experience (20, 21). All participants were recruited through referrals from the study team’s professional network, the WUSM Department of Neurosurgery, and snowball sampling. Neurosurgeons were also recruited using publicly available contact information from websites of GBM-focused treatment centers and nonprofit organizations (e.g., professional societies, private foundations). Before recruiting neurosurgeons, study team members reviewed available professional information (e.g., areas of focus or journal publications) to verify that potential neurosurgeons treat GBM patients.

Patients were confirmed to have a GBM diagnosis by their clinical team at Washington University prior to study referral. Eligible caregivers needed to have provided support to a patient diagnosed with GBM, such as organizing and attending healthcare appointments, serving as a healthcare proxy, and assisting with tasks of daily living. These individuals were typically family members (e.g., spouse/partner, parent, child) referred by the clinical team or a patient. When a patient and caregiver were not able to participate as a dyad, patients or caregivers were able to participate individually.

Our initial recruitment goal was 15 participants in each stakeholder group. Several invited patients and caregivers could not participate due to being too sick or balancing numerous commitments (e.g., radiation, chemotherapy, work, childcare). Despite not reaching our initial recruitment target for all groups, our sample of 13 patients and 14 caregivers remains justifiable, as a sample size of 12 has been shown to be sufficient for reaching thematic saturation in qualitative interviews, particularly interviews that employ purposive sampling to ensure adequate representation of the target population (22–25).

Potential participants received up to four emails and two phone calls to deliver recruitment, eligibility, and consent information. Recipients were invited to schedule an interview, and scheduled participants received a confirmation email with a Zoom link and copy of the interview questions. This research was approved by the Washington University in St. Louis Human Research Protection Office (IRB ID #202411172).

### 2.2 Data collection

Interview guides for all three stakeholder groups were informed by literature on ethical considerations for artificial intelligence (AI) in clinical decision-making, as well as literature on patient, caregiver, and healthcare provider perceptions of using AI to support clinical decision-making for serious or terminal illnesses (26–28). In order to provide a case reference for participants, interviews began with background information about a real-world ML model currently under development by the WUSM Department of Neurosurgery, but participant responses to interview questions were reflective of perceptions about ML in GBM broadly. The ML model used as a case example is designed to predict the prognosis (i.e., life expectancy and quality of life) of GBM patients and inform surgical decisions for neurosurgeons. The study team collaborated with the ML model development team to ensure terminology in the interview guide and background information were appropriately worded. Further information about the model’s architecture, dataset, and training and validation procedures are available in existing publications (9, 29).

Background information presented to participants stated that the ML model would use data from a patient’s resting state fMRI and other health data (e.g., age and brain tumor size) to predict a patient’s prognosis. The presentation shared the ML model’s current accuracy for predicting prognosis (i.e., 90% for life expectancy, 94% for KPS) and explained that doctors could review information generated by the ML model to help them make decisions about how best to treat a patient’s brain tumor (9, 29). Interview guides, background information, and codebooks are publicly available through the Qualitative Data Repository (QDR) at Syracuse University (30).

Three study team members conducted interviews, including the principal investigator (TM), project manager (MP), and graduate research assistant (OB). All interviewers had prior experience conducting qualitative research. Prior to data collection, interviewers were engaged in interview guide development and established a protocol to standardize procedures. Interviewers participated in peer-interview shadowing to finalize interviewer training and met weekly throughout the study to discuss the progress and quality of data collection.

Interviews were conducted and recorded using Zoom and were professionally transcribed prior to analysis. Interviews began with verbal informed consent and presentation of background information. Participants were permitted to ask questions about the ML model after being presented with background information and throughout the interview. Patient and caregiver interviews lasted 60 min each. Neurosurgeon interviews lasted 30 min to accommodate busy schedules and encourage participation.

In addition to questions about their experiences with GBM diagnosis and treatment, participants were asked about perceived benefits and concerns of using the ML model to: (1) predict patient prognosis, and (2) make surgical decisions for treating the patient’s brain tumor. Patients and caregivers were also asked if their attitudes would change if the ML model was no longer experimental, but well-established in current medical practice. Participants were asked additional questions, such as what knowledge and skills they think neurosurgeons need before using the ML model, information patients and caregivers should know about the ML model, how disagreements between the neurosurgeon and the ML model should be handled, and how the ML model may impact the doctor-patient relationship; these findings will be reported in forthcoming papers. Interviews concluded with demographic questions, presented in Table 1. Participants received a $60 gift card for completing an interview.

**Table 1:** Demographic characteristics of participants (*N* = 42).

Demographic	Patients (*n* = 13)	Caregivers (*n* = 14)	Neurosurgeons (*n* = 15)
Age			
30–39 years	2 (15%)	1 (7%)	6 (40%)
40–49 years	2 (15%)	2 (14%)	5 (33%)
50–59 years	1 (8%)	4 (29%)	2 (13%)
60–69 years	7 (54%)	6 (43%)	2 (13%)
70–79 years	1 (8%)	1 (7%)	0 (0%)
Gender identity			
Male	9 (69%)	3 (21%)	13 (87%)
Female	4 (31%)	11 (79%)	2 (13%)
Racial identity			
Asian	0 (0%)	0 (0%)	7 (47%)
White	13 (100%)	14 (100%)	8 (53%)
Ethnicity			
Hispanic or Latino	0 (0%)	0 (0%)	1 (7%)
Not Hispanic or Latino	12 (92%)	14 (100%)	14 (93%)
Prefer not to answer	1 (8%)	0 (0%)	0 (0%)
Highest level of education*			
High school	2 (15%)	2 (14%)	
Some college	3 (23%)	3 (21%)	
Associate’s degree	1 (8%)	0 (0%)	
Batchelor’s degree	3 (23%)	6 (43%)	
Master’s degree	2 (15%)	3 (21%)	
Doctoral degree	2 (15%)	0 (0%)	

*This question was not asked in the neurosurgeon stakeholder group because it was independently verified that they had a doctoral degree.

### 2.3 Data analysis

Thematic saturation was reviewed and discussed weekly by the study team during data collection, with no new themes appearing in later interviews (25). Data analysis involved a structural and inductive thematic approach using Dedoose qualitative data analysis software (31). Codebook development involved the principal investigator (TM) and two interviewers (MP & OB), beginning with a review of participant transcripts and discussing common themes observed. Separate codebooks were developed for neurosurgeons, patients, and caregivers, but codebook structure was similar across all groups. Codebooks were organized using both structural and thematic coding, capturing participants’ overall responses to each question and the themes within each response. Additional thematic codes captured perceived benefits and concerns related to ML model use expressed throughout a transcript. All codebooks included a “new idea or other important information” code to capture unique thematic ideas not reflected in the codebook. Once each codebook was drafted, two coders (MP & OB) tested the codebook on a subset of transcripts. The study team met to discuss and resolve discrepancies and refine the codebook before coding remaining transcripts.

All transcripts were blind-coded by a primary (OB) and secondary (MP) coder. The study team held regular meetings to assess interrater agreement, resolve coding discrepancies, and reach consensus among coders to establish 100% interrater agreement for each transcript (32). Code presence was counted to understand the number of participants whose interview reflected that code. The study team reviewed all coded data to generate summaries of code content and identify illustrative quotes.

## 3 Results

### 3.1 Sample characteristics

#### 3.1.1 Patients and caregivers

Many patients (10/13) and all caregivers (14/14) discussed the emotional toll of GBM diagnosis and treatment. Patients primarily recalled feelings of shock and overwhelm in the short period between diagnosis and surgery, often noting that GBM was not the diagnosis they expected to receive. Caregivers reiterated the shock felt at the point of diagnosis and the uncertainty throughout treatment, such as not knowing whether or how effective a treatment plan would be. Caregivers also expressed feeling grief while witnessing their loved ones’ treatment side effects or brain tumor symptoms.

“*…we really didn't know how long he had…Maybe they can't tolerate the chemo or maybe he hadn't tolerate the radiation…It was really difficult from the start, is all I can say, just emotionally and not knowing exactly how this was gonna play out.”* Caregiver 1

“*It was shocking because the week that I went in for the brain tumor, I actually thought I was having a stroke…I realized at a restaurant that Saturday night that I couldn't get my wallet out of my back pocket. My wife thought I was having a stroke, so we drove to [the hospital] thinking the whole time it was a stroke, and here they come back with the diagnosis, it's a brain mass. It's a little shocking.”* Patient 1

While the diagnosis and treatment of GBM was harrowing, some patients (6/13) and caregivers (2/14) shared positive experiences. They expressed gratitude for their support systems, trust in their healthcare providers, celebration of victories, and feelings of hope.

“*I've never really stayed in a hospital …I was able to get in on a Monday, have surgery Wednesday, and leave Friday, which was a pretty quick turnaround. Got connected with an awesome surgeon and awesome team there. It's a very positive experience*.” Patient 2

*“He is responding to it [chemotherapy] very well. The tumor is shrinking. He feels pretty good. People look at him and have no idea. They can hardly see the scar from his brain tumor, and he is—doesn’t look sunken in and sick all the time like most cancer patients look. He's doing well.”* Caregiver 3

When asked about the support systems they relied on during GBM diagnosis and treatment, most patients (9/13) discussed support from a primary caregiver, typically a spouse or partner. They expressed gratitude for both physical and emotional support throughout treatment, daily living, and healthcare decision-making. Many patients (8/13) also sought support from secondary caregivers such as family or friends, as well as members of their faith community or GBM support groups.

*“Just bein’ here for me. My wife, she's does everything for me. I can't describe how much she does for me; how much I appreciate it. My daughter, she goes to most of my doctor's visits, just to stay up to date, and her input, and questions, that type of thing. It's just knowin’ they're there for me means a lot, big support.”* Patient 4

*“My preacher from our church came in several times. I was just like, "Eh, we got this. God's gonna take care of me." I guess that was the reason I wasn't so upset …Maybe I just wasn't processing that this could be a death sentence…I just knew there were so many people praying for me, that helped so much. It was amazing.”* Patient 5

Equally, patients (9/13) acknowledged the support of their neurosurgeon, particularly when patients were trying to understand their diagnosis and make decisions about their treatment plan. Some patients (3/13) also mentioned support they received from other members of their healthcare team, such as nurses, radiologists, and oncologists. In addition to empathy and sensitivity from their healthcare providers, these patients emphasized the high level of trust they placed in their neurosurgeon and other healthcare providers.

*“Really, my view goin' into this was, I don't wanna tell the doctor what I'm gonna not do and can do. They're the experts. We have a great staff here in [City 1], and just listen to what they think is the best choice moving forward and really just take their direction and have confidence in the team.”* Patient 2

Caregivers described their role in supporting their loved one with GBM as organizing and managing healthcare appointments for the patient (12/14), often involving transportation or accompaniment to appointments, and helping the patient with tasks of daily living such as managing medications, household chores, and childcare (9/14). Caregivers also served as important advocates for the patient by helping them understand complex health information from providers (7/14), as well as communicating patient needs and concerns with the patient’s healthcare team (4/14).

“*She [the patient] has a two-year-old and four-year-old. I had to go to her house every single day and get the one ready for preschool and cook and clean and do everything for her …I have to constantly remind her, even though she has a notebook and a calendar and Post-it notes, it's—I sent her a text message this morning, "Don't forget about your meeting, " because she forgets to look at her calendar or her Post-it notes.*” Caregiver 6

“*I drove him to all of his doctor’s appointments, and I went in with him. As he was having problems, I would put ‘em in my phone and then print ‘em out, so we knew what to talk to the doctor. When he’d go in, they’d ask him, “So how you doin’?” He’d say, “Oh, I’m fine.” “No, you’re not.” You’re fine right at the moment but no. Yeah, it was difficult*.” Caregiver 2

#### 3.1.2 Neurosurgeons

All neurosurgeons (15/15) discussed their experience treating GBM patients in their clinical practice. Four neurosurgeons reported other professional activities related to GBM treatment, such as having a residency or postdoctoral focus on GBM, serving as director of brain tumor centers for GBM, or conducting research and development of medical devices used in GBM treatment. The majority of neurosurgeons (11/15) also treated other types of brain tumors such as benign tumors or skull base pathologies.

Most neurosurgeons were within the early-career (i.e., 10 years or less, 6/15) and mid-career (i.e., 11–25 years, 6/15) stages of their clinical practice, with three surgeons having more than 25 years of clinical experience. When asked about their familiarity using AI tools at work, three neurosurgeons reported having no experience using AI, five had only limited general experience using AI in clinical contexts (e.g., scribing, clinical notes, writing assistance), and seven had used AI to inform clinical decisions for patient care (e.g., generating prognostic information, interpreting radiographic data).

### 3.2 Prognosis prediction

In what follows, we present the perceived benefits and concerns of using an ML model for GBM prognostication among patients, caregivers, and neurosurgeons. Code counts representing the number of transcripts where each theme emerged is presented in Table 2.

**Table 2:** Perceived benefits and concerns of using a ML model to predict patient prognosis among patients, caregivers, and neurosurgeons.

Theme	Patients (*n* = 13)	Caregivers(*n* = 14)	Neurosurgeons(*n* = 15)
Benefits of the ML model to predict prognosis			
Guide neurosurgeon decision-making	7 (54%)	3 (21%)	6 (40%)
Expands the scope of possible information to be taken into consideration	6 (46%)	6 (43%)	5 (33%)
Guide patient decision-making	5 (38%)	6 (43%)	4 (27%)
Increase patient understanding of their diagnosis or quality of life	5 (38%)	7 (40%)	0 (0%)
Provide information about different treatment pathways	5 (38%)	4 (29%)	0 (0%)
Concerns of the ML model to predict prognosis			
Insufficient accuracy and applicability of model output	6 (46%)	6 (43%)	10 (67%)
Potential loss of hope for patients	5 (38%)	7 (40%)	1 (7%)
Reservations about experimental nature of model	5 (38%)	6 (43%)	0 (0%)
Potential reservations about or distrust in model	5 (38%)	5 (36%)	0 (0%)
Potential neurosurgeon over-reliance on model	1 (8%)	2 (14%)	5 (33%)
Model could cause more confusion for patients and families	1 (8%)	1 (7%)	3 (20%)
Model could bias patient perceptions of treatment plan	1 (8%)	0 (0%)	1 (7%)

#### 3.2.1 Patients

##### 3.2.1.1 Perceived benefits

All 13 patients mentioned at least one perceived benefit to using the ML model to predict GBM patient prognosis. Patients (7/13) primarily discussed the assistance the model could provide in neurosurgeon decision-making, helping surgeons to better understand the risks of different surgical approaches, leading to better outcomes for individual patients. A common perception (6/13) was that the model could improve surgical decision-making by taking into account a wider scope of patient data that a neurosurgeon might not have been able to consider otherwise. These patients felt that having more data about an individual patient, as well as a library of information from similar patient cases, could serve as an additional credible source to improve the precision of surgical planning.

“*I guess a benefit would be that it could maybe besides just the patients at one medical center, it could pull information from a greater number of patients. Then you'd have a bigger group to pull from. Maybe that would provide even more accuracy with a bigger sample size. I guess just the benefit of having information that you wouldn't otherwise have.”* Patient 7

Similarly, patients (5/13) felt that prognosis predictions from the ML model could provide beneficial information about various treatment pathways and help patients choose a path that best aligns with their priorities (e.g., quality vs. quantity of life). Patients equally (5/13) mentioned that the model could increase understanding about their predicted quality of life and how their tumor and treatment plan could affect brain function over time. For all these reasons, patients (5/13) felt that the ML model could support planning and decision-making for patients and families, providing them with individualized information to select treatment pathways and end of life care plans tailored to the patient’s goals.

*“I would definitely be open to any new technology, including this, to guide treatment…I don't think my focus would be necessarily on life expectancy. I would definitely wanna understand how that could help with treatment, quality of life, things like that…is there anything I can do to help the process health-wise on my end, whether it be exercise, diet, whatever? Then if there's different treatment options that go down different paths, what can lead to a better quality of life and focusing on that rather than a number that I'm tryin' to focus on out into the future.”* Patient 2

##### 3.2.1.2 Perceived concerns

Nearly all patients (12/13) discussed one or more concerns about using the ML model to predict prognosis. The most common concern among patients (8/13) was the potential for the model to produce erroneous predictions. They emphasized the need for diverse patient datasets, including diversity of tumor type, size, and patient age to continuously train and refine the model.

*“I guess a reservation would be there’s just so many factors that it seems like would go into that [prognosis prediction]. Especially from what I understand about brain tumors because there’s many* var*iations of types of tumors and then size of tumors and then just the age of the patient and how it affects different people.”* Patient 7.

Patients (5/13) further discussed reasons why they may distrust prognosis prediction generated by the ML model. Some of these patients felt that AI or computers in general were less credible than human reasoning. They emphasized the importance of consulting their neurosurgeon’s opinion when interpreting the model’s prognosis prediction. Others articulated how their faith beliefs drove their understanding of their life expectancy, expressing that only God knows when the last day of their life will be, rather than predictions by a computer or neurosurgeon. One patient mentioned that they could lose trust in their neurosurgeon if they thought their surgeon was relying too heavily on the model, but also felt this reliance was unlikely.

“*I'm old-fashioned. I'm old-school type guy. Well, I'm old. That explains a lot right there. I don't know if there benefits for me anyway ‘cause I wouldn't believe a computer. I'd wanna talk to the doctor, ask questions, his opinion, that type of thing*…*My opinion it’s, doesn't matter who you talk to, computer, doctor, it's a guess. There's only one person knows the number of days you have, and he's upstairs. All my life I believe your days are numbered.*” Patient 4

“*Now, what I would be concerned by would be a doctor that falls in love with technology and says, “Oh, the algorithm, based on artificial intelligence, says you’re going to live 5.6 years.” I can’t imagine a neurosurgeon is going to become tethered to technology like that.”* Patient 8

While patients were concerned about the accuracy and credibility of the model, several (5/13) felt that their confidence in the model may increase over time as it is refined and becomes more established in clinical practice. This sentiment about increased confidence was in contrast to the current experimental state of the ML model’s development.

*“I would probably trust it more the more that it's been vetted and that kind of thing. Yeah…After hearing about studies that have been done for a long time and knowing that things work well, I usually would be more apt to trust that a little bit more.”* Patient 7

*“I think it would make me feel better if it was established and it had a success rate, I guess, so that you could—that would be one of the questions that I would ask. Okay, you wanna use this program. That's fine. What is the success rate?”* Patient 9

Several patients (5/13) also considered the emotional impact of receiving information about their prognosis from the ML model. They emphasized that information, particularly about life expectancy, is overwhelming and very difficult for patients and families to process. These patients highlighted the importance of reasons to have a positive outlook in their situation. They expressed concern that the model’s survival prediction could introduce a level of determinism that leads patients to feel hopeless or lose a sense of autonomy.

“*My concern for my fellow patients is that they would put too much credibility to it. As a consequence, right, it could cause depression, feelings of hopelessness, have them give up on what could be helpful sorts of treatments. I think it lends too much credibility to the concept of life expectancy*.” Patient 8

One patient mentioned that, amidst the stress of a GBM diagnosis, prognosis prediction from the model may only add a layer of confusion for patients and families. Alternatively, another patient considered how prognostic information generated by the model could inflate patients’ sense of hope because patients would know there is a chance the model’s prediction is inaccurate. Knowledge of possible inaccuracies could benefit patients’ motivation to continue with their treatment plan, but this knowledge could also cause patients to neglect certain realities of their situation.

*“Well, [the concern is] that everybody would take it as the God's honest truth or a gospel truth, I mean. There's always an exception here or there. Again, it's just an idea of what should be, what computer's telling it is the possibilities. Some people beat the possibility, beat the odds, some people don't beat the odds. It always gives you hope.”* Patient 1

##### 3.2.1.3 Unique perceptions

One patient stated concerns about the privacy and nature of sensitive and personal patient data used by the model. This patient was concerned about patient data being used for reasons other than predicting prognosis or supporting surgical decision-making by the ML model, such as negatively impacting health insurance rates.

“*I know with a lot of this, sometimes there's concern about personal data, PII type of stuff. I honestly don't have a whole lot of concerns about that. I think I would trust the folks doin' this…Now if for some reason down the road that had impacts where companies were allowed to take that data and charge different health insurance rates or something like that, maybe that's a factor, but I haven't encountered that in my life today. I would say that's the one thing, is who is gonna get access to this, not for any concern of using as part of a model, but for any pricing impact down the road to things you might need through different products*.” Patient 2

Another patient felt that a benefit of the model was that it removed the influence of emotions in difficult prognostic conversations. This patient expressed anxiety about family members or healthcare providers attempting to spare patients’ feelings when discussing prognosis. They saw security in the model’s ability to present this information regardless of how it might be received by patients.

*“Well, I guess it takes the emotions out of it. You don’t have a doctor tiptoein’ around maybe tellin’’ you the truth ‘cause I know I have been worried about that in the past because when I first found out, I immediately think of my kids and be like, “You guys have to save my life.” I would get upset and then be afraid that they weren’t gonna be as straightforward with me because they have some hysterical woman in their room. I guess that would be good to take the human aspect out of it, and I don’t know. I think it’s great that we just have another tool. There’s next to zero treatment for this particular cancer.”* Patient 6

#### 3.2.2 Caregivers

##### 3.2.2.1 Perceived benefits

A significant majority of caregivers (13/14) acknowledged at least one benefit to using the ML model to predict patient prognosis. Specifically, seven caregivers discussed how the model’s output could improve their understanding of the patient’s projected quality of life. Caregivers felt this knowledge would assist them in preparing for subsequent phases of patient care, comprehending potential side effects and impact on daily life, and finding ways to maximize the enjoyment of the patient’s remaining time.

“*I think that would be fantastic…To talk about quality of life would be huge. We touched on that some, but basically, we were told every patient's different. I get that, but to be able to predict it with that kind of accuracy I think would be fantastic*.” Caregiver 1

Similarly, nearly half (6/14) of caregivers suggested that the model could take into account a wider scope of information about the patient than the neurosurgeon alone. Caregivers (6/14) also highlighted that model-informed insights could enable patients to make well-informed decisions regarding their care plan.

*“Knowing. Honestly, the knowledge. Again, I think it would've changed a lot of what we would've done. I don't know that we would've had surgery…I don't know we would've put him through that. It didn't change the outcome very much…At the time, we thought it was going to extend his life another year. Had we had this program, I—and the program, again, told us it was—it wouldn't have changed anything, we wouldn't have put him through it.”* Caregiver 10

Furthermore, caregivers (4/14) expressed that prognostic details would offer a clearer understanding of potential treatment pathways, empowering patients and families to choose treatments that optimize quality of life. Some (3/14) noted that the model output could support doctors in treating the patient, particularly when used in conjunction with the physician’s clinical experience, leading to more informed recommendations based on individualized patient data.

*“I personally, I would support it. I would be excited about it because I've learned over this whole 11 months of this, standard of care is not a one-stop shop. It doesn't fit everyone. I would love to hear how they can customize that to my husband with all of—everything about him. Any medicines he's on, anything like that. Prior to this, what was his health like, prior to this and customize that. I think the way I understand it, this is something that this computer program could, more, drill in on him, not that one big umbrella of standard of care.”* Caregiver 4

##### 3.2.2.2 Perceived concerns

Caregivers (13/14) also expressed several concerns regarding the use of the ML model to predict prognosis for patients with GBM. A primary concern identified by caregivers (7/14) was the potential for the model-generated prognosis prediction to leave patients feeling hopeless, especially when the model suggested a shorter life expectancy than the patient had anticipated.

“*If it was my wife finding out—the patient finding out about this and then it's wrong—let's go the other direction. Maybe it says they're gonna live two years, and they thought they're—or one year, and they thought they were gonna live two years. It makes them stop fighting, right?”* Caregiver 11

Furthermore, caregivers (6/14) were apprehensive about the potential for errors in the model’s prognosis prediction and the consequences of incorrect predictions for a patient’s wellbeing and treatment plan. They urged neurosurgeons to be cautious in their interpretation of the model’s output. Because GBM can significantly differ from one patient to the next, caregivers emphasized the importance of ensuring diverse cases are represented in the model’s training data to foster confidence in the model’s accuracy.

“*Well, computers, there's always errors, right? It could be wrong. They could’ve transposed a number or focused heavily on one aspect and not weighed in other aspects of the studies. That could be human error. That could be computer error. It could be the person that's inputting the information error. There's always a chance of error, where they would say err on the side of caution. I think that happens in everyday life*.” Caregiver 3

Six caregivers indicated that they would feel more comfortable with the model if it were commonly used in medical practice and had achieved greater data validation and endorsement from medical professionals over time, compared to a model in experimental stages of development. They believed a well-established model would enhance trust in the accuracy and reliability of the model’s output.

“*If it's been running for a good year and they're seeing comparative results that are definitely—that are showing the right outcomes, I would be willing to accept that. 'Cause pretty much what I've looked at, sometimes you put the information in the computer, it's exactly what the doctor has said.”* Caregiver 12

Some caregivers (5/14) expressed skepticism of AI in general, and discussed how their personal experiences and media portrayal of AI apply to the current ML model. In addition to concerns with inaccuracies in the model training data or output, caregivers worried that the use of AI in healthcare settings may reduce the human touch in patient care, which they deemed crucial.

*“If someone said to me, "…you're going to be working solely with an AI program", I would definitely not do it, but knowing that I'll always have human contact and a doctor translating the AI information and using the AI information to help him, I would be in favor of it.”* Caregiver 3

Two caregivers emphasized the importance of neurosurgeons’ transparency in explaining the nature of their reliance on the ML model for decision-making. These caregivers were primarily concerned with neurosurgeons solely relying on the model to draw meaningful conclusions about a patient’s prognosis without consulting other doctors or drawing from their clinical experience. Caregivers felt that over-reliance on the model could erode the human element of patient care or lead to mistrust in the neurosurgeon, especially if patients and caregivers perceived the model’s prediction to be incorrect.

“*The part that's scarier to me is if the physicians are using this data to make their prognosis or their treatment plan and not informing the patient or the family that they're using this and it's wrong. Then I don't trust the physicians anymore and that's a problem*.” Caregiver 11

Further emphasizing the importance of doctor-patient communication, one caregiver highlighted that the model’s output could potentially confuse patients and families, particularly those less familiar with AI. If the neurosurgeon’s use of the model and interpretation of its output was not accessibly explained to patients and families, they noted this could add to their stress and anxiety rather than alleviate it.

“*My mother, for example…She doesn't understand how [to use basic technology], and she doesn't care to learn how…I think about brain tumors and the glioblastoma. That's that age. I think that might be a barrier. Some people who would use it, I think their children—their Millennial children, their Gen Z children, their Gen X children—I think would be 100 percent on board. I don't think there would be a barrier there*.” Caregiver 10

##### 3.2.2.3 Unique perceptions

One caregiver noted that trust in the experimental algorithm will vary across individuals in that some people will be inherently more open to and trusting in an experimental model than others.

*“My partner and I are always in the same meetings with the doctors, right? My partner tends to be a little bit more pessimistic, and he was always that way. [Laughter] I’m a little more optimistic, so I think, depending upon the personality, for me, if someone said, ‘This is experimental,’ I would be able to acknowledge that and own that, and be like, ‘This is experimental. There isn’t a lot of statistical information to back this up.’…I’m gonna take it with some healthy skepticism because it’s experimental. I think other people, if their personality is a little more—they’re gonna not hear the experimental part and just be like, ‘This is the answer because technology, which is all-powerful, has told me this.’”* Caregiver 13

Two other caregivers illustrated the idea that individuals will vary in their perceptions about the emotional nature of the model. One caregiver mentioned a benefit of the model lacking an emotional component in prognostic conversations, potentially leading to unbiased information delivery. In contrast, the other caregiver expressed concern that the absence of emotion in the model would feel “cold,” lacking the human touch in they experience with their healthcare providers.

*“I would think it's non-biased, and that would be—it's a computer. It has no feelings. Yeah, I think it would be a non-biased opinion.”* Caregiver 6

*“It [the model] feels a bit cold. The whole predicting feels very cold. I understand why it has to be done, and I understand we asked for the information, right, ‘cause you wanna know, but I think it can take away the— it could take away some semblance of the real lived experience and staying focused and on what is happening. It is so hard to continue pushing away the—‘cause the information’s coming in all the time.”* Caregiver 8

#### 3.2.3 Neurosurgeons

##### 3.2.3.1 Perceived benefits

Most neurosurgeons (10/15) articulated at least one potential benefit to using the ML model to predict patient prognosis. Neurosurgeons noted that the ML model output could assist neurosurgeons (6/15) and patients (4/15) in making treatment decisions. Neurosurgeons felt that, when combined with clinical expertise, model output could help neurosurgeons devise the best possible treatment plan for each patient. Similarly, neurosurgeons felt that having prognostic information from the model could help patients and families make decisions about short- and long-term care (e.g., undergoing chemotherapy, radiation, or planning end of life care) based on the predicted survival and quality of life.

*“…it's [the model] helpful and beneficial both for the patient, but obviously for the surgeon as well. Not only from an operative standpoint, but also from a outcomes and a planning standpoint*.” Neurosurgeon 1

Neurosurgeons (5/15) also acknowledged that the model would be able to take into account a wider scope of patient information than the neurosurgeon alone. This broader scope of data could be used in tandem with a neurosurgeon’s clinical experience to account for how comorbidities or lifestyle factors might affect a patient’s treatment plan. Neurosurgeons thought this hybrid approach of model output and surgical expertise could enable more accurate prognosis prediction.

“*I think there's a lot of potential for prognostication based on large data incorporating algorithms and whether that includes radio graphic findings, genetic findings, patient characteristics, surgical factors. I think a lot of that it needs to still be improved beyond just model building, but there's huge, I think, amounts of data that's not being analyzed clinically and is taken on a much more qualitative assessment when reviewing things like MRI scans or patient comorbidities, rather than a more quantitative or data-driven approach, so I'm sure it will have a larger and larger impact on how we counsel patients and how our treatment plans are determined moving forward.*” Neurosurgeon 2

##### 3.2.3.2 Perceived concerns

Many neurosurgeons (13/15) also noted concerns with model use. Predominantly, neurosurgeons (10/15) were concerned about the quality of data used to develop the model, including both data accuracy and the diversity of training data sources. Their concerns included the quality and rigor of data used to train the model and how inaccuracies or biases in the data might affect real-life patient outcomes.

Because the ML model does not predict prognosis with 100% accuracy, surgeons were concerned about patients who fall into the inaccurate percentile of prognosis prediction cases and whether this could negatively impact their treatment plan. Further, neurosurgeons stressed that GBM is a heterogenous disease. Because of this, neurosurgeons shared concern that the ML model would not be able to predict prognosis in a way that captures the individuality of each patient if it was not trained on a dataset involving a comprehensive range of patients with different demographic characteristics, tumor types, and treatment plans.

“*I think that all of these machine learning algorithms are really heavily influenced by the data that you put in. The teaching cohort or the discovery cohort really impacts what goes in. There's a lot of heterogeneity [among GBM patients], and so even a large population of 130 patients does not fully encompass the heterogeneity.”* Neurosurgeon 3

Several neurosurgeons (5/15) acknowledged the risk of neurosurgeons relying too much on the ML model to predict patient prognosis, rather than using it as a supplementary tool for their clinical judgment and experience. They stressed the importance of using the model to support but not replace neurosurgeon decision-making, similar to how radiographic imaging tools support decision-making. Two of these neurosurgeons cautioned that over-reliance on the model could cause patients to lose crucial trust in their surgeon or care team.

*“…I'm a little worried about someone taking this information and making decisions which, again, are imperfect, telling somebody they've got 18 months to live or something, when we know we're not perfect at predicting that.”* Neurosurgeon 4

Similarly, three neurosurgeons cautioned that the model’s prognosis prediction might cause unnecessary confusion for patients and families if complex output information were made available to them without proper expert-informed discussion of what the survival and functional outcome information means for them (e.g., via patient portal). For instance, if the model predicted short-term life expectancy for a patient, one neurosurgeon expressed concern that this could negatively impact patient decision-making and how they perceive their treatment options.

“*If the tool takes inputs that are too complex for a patient to really understand, but the tool is available for patients to use, then I think there's a risk that patients will be misled one way or another about their prognosis. I think we just have to be thoughtful about who gets to use it, how they get to use it. What are the inputs and what are the outputs? What is the ability of the user to interpret accurately all of those pieces of information?*” Neurosurgeon 5

Finally, one neurosurgeon was concerned that sharing the results of the ML model with the patient could introduce a level of determinism in the patient’s perception of their life expectancy and quality of life, causing them to lose hope or become disengaged with their treatment plans.

“*The major risk is converting one of the things that we do as physicians and surgeons is providing hope to the patients that come to us when they've heard and they've seen on the internet that there is no hope. Incorporation of these algorithms into the clinical practice and the discussions that we have with the patients should be done very carefully because there is an element of determinism in these algorithms. X plus Y equals Z, and that's not always the case.”* Neurosurgeon 6

##### 3.2.3.3 Unique perceptions

Two neurosurgeons acknowledged that the true impact of the model on GBM treatment will remain unknown until it has been used by various neurosurgeons across multiple treatment centers. They also noted that the model will require ongoing refinement. One neurosurgeon felt that using an ML model was no different than any other technology currently used by neurosurgeons in everyday practice.

*“I think, at the end of the day, this will be validated in real world how it affects the outcomes. Obviously, I think even if it gets initially approved and gets implemented in clinical practice, I think a few years of using it will probably tell us and further studies will tell us whether this is actually helping or whether it's actually withholding important treatments from patients”* Neurosurgeon 6

*“It’s like any other technology. It’s like any other study. There’s nothing special about it. It needs to be well done so its conclusions can be sustained, but in terms of using MRIs and analyzing ways to predict outcomes, it’s a tale as old as time. There’s nothing new about that.”* Neurosurgeon 7

### 3.3 Surgical decision-making

In what follows, we present the perceived benefits and concerns of using an ML model to inform surgical decision-making. Code counts representing the number of transcripts in which each theme emerged is presented in Table 3.

**Table 3:** Perceived benefits and concerns of using a ML model to make surgical decisions among patients, caregivers, and neurosurgeons.

Theme	Patients (*n* = 13)	Caregivers (*n* = 14)	Neurosurgeons (*n* = 15)
Benefits of the ML model for surgical decision-making			
Guide neurosurgeon decision-making	8 (62%)	6 (43%)	3 (20%)
Guide patient decision-making	3 (23%)	2 (14%)	2 (13%)
Facilitate communication between neurosurgeons, patients, and families	2 (15%)	2 (14%)	3 (20%)
Expands the scope of possible information to be taken into consideration	2 (15%)	1 (7%)	2 (13%)
Provide information about different treatment pathways	1 (8%)	1 (7%)	0 (0%)
Concerns of the ML model for surgical decision-making			
Reservations about experimental nature of model	6 (46%)	6 (43%)	0 (0%)
Potential neurosurgeon over-reliance on model	2 (15%)	3 (21%)	3 (20%)
Potential patient reservations about or distrust in model	2 (15%)	2 (14%)	0 (0%)
Insufficient accuracy and applicability of model output	0 (0%)	0 (0%)	4 (27%)

#### 3.3.1 Patients

##### 3.3.1.1 Perceived benefits

The vast majority of patients (10/13) mentioned at least one benefit to using the ML model to inform surgical decisions. Many of these patients (8/13) felt that neurosurgeons could use information from the model alongside their professional experience to narrow down the best possible treatment approaches. Two patients stated that having the model available to draw from diverse data sources could help precisely predict post-operative outcomes for patients or anticipate risks associated with surgery.

“*Modern medicine is always gonna evolve to help and get better. AI might be the next best thing that's gonna happen, because…you're limiting the things that can go wrong. You can put more information in, and they can help determine what should happen, or what maybe not be the best case. That just—that's with all the information you're able to pile into the computer and let it filter out and figure out, and have it work. Technology's good, to a point*.” Patient 3

Some patients (3/13) felt that the model could provide one more source of information to help patients make decisions about their care, such as whether to seek care at a different hospital that offers the patient’s preferred surgical option or whether to undergo surgery at all. Similarly, two patients thought that the recommendations from the model could facilitate more productive conversations with their neurosurgeon about selecting a treatment pathway that best suits the patient’s priorities. One of these patients mentioned that the model could help them understand the benefits and risks associated with certain surgical approaches.

“*What might be a good way to tackle it is, what is the surgeon's pre-AI thoughts, and then what are their thoughts with the AI, and what are the risk factors? How extreme are those risks? 'Cause obviously, if you're talking about a more aggressive approach, that quality of life impact of going too far and changing brain function's a really big deal. You can't reverse something like that. That one's a little bit more concerning, but I think you could have a conversation of still trusting the doctor and what is the recommendation and just trying to understand what goes into that*.” Patient 2

##### 3.3.1.2 Perceived concerns

Patients (7/13) discussed fewer overall concerns with the ML model being used to inform surgical decisions. The primary concern they expressed was that the model was experimental and still under development (6/13). While these patients did not say that the experimental nature of the model would make them reluctant to have it used, it would make patients feel more comfortable if the model was well-established in standard clinical care.

“*I think it would change, because I think knowing that it was well established, I would feel like it had been utilized and had been seen to work. I would feel like they wouldn't use something that they didn't think was working well. It must be something that they feel is really helpful to them in the next steps.”* Patient 7

Two patients emphasized that they wanted their neurosurgeon to use the model in conjunction with their expertise, without relying too heavily on the model’s output to make surgical decisions. Two other patients echoed the sentiment that they did not trust the model’s recommendation to be the only piece of information taken into account when making surgical decisions; they expected their neurosurgeon to exercise their clinical judgment when interpreting the model output and making recommendations to patients.

“*If I trust my doctor, I would want to do what my doctor wanted, not what the computer told him to do. I would think that he knows better. I would like that he's reaching out to other avenues to try to gain more information. I would like that. I would think his expertise would be the, for me, the biggest thing. That's puttin' a lot of pressure on that man*.” Patient 5

#### 3.3.2 Caregivers

##### 3.3.2.1 Perceived benefits

Caregivers (8/14) highlighted several potential advantages to using the ML model to inform surgical decisions. Nearly half (6/14) of caregivers noted that the model could assist neurosurgeons in being more precise and developing the best treatment plan for a given patient. One caregiver noted that the model’s ability to consider a wide range of individual patient data could improve the quality of treatment recommendations.

“*Knowing our own experience with the brain surgeon, who was wonderful, it was just empowering them more to make decisions…A brain surgeon saying to me, “We’re gonna use this experimental AI to make some decisions about which method, what we’re gonna do with surgery, or not surgery,” I would be completely comfortable with that*.” Caregiver 13

“*I can accept that [using the algorithm to inform surgical decisions], and I can see why it would be helpful, potentially, in a variety of different ways for the doctor to have that input, especially if they’re waffling on something, or if it’s just a close, just like, “Gosh, it could go a couple of different ways. There’s so much at stake. Why not use this data?”* Caregiver 8

Similarly, a couple of caregivers (2/14) felt that the model could enable patients to make more informed choices about how to handle their diagnosis. Two other caregivers thought the model output could provide a clearer framework for discussing the risks and benefits of different treatment options, fostering better communication between neurosurgeons, patients, and caregivers. Another caregiver mentioned that the model could help patients better understand different treatment options, benefiting their overall decision-making process.

*“…I would feel, more, that this is more customized to my loved one, is having this program. Because we already know what's out there, but what else can they add? I think…this would give us something else to think about when, you know, we're making decisions, this would be a tool that we would use…If you could provide that data that, hey, this does work, we've got live cases, we've got real people. It's not just statistics, it's live cases, live data. I'd be all for it.*” Caregiver 4

##### 3.3.2.2 Perceived concerns

In addition to perceived benefits, caregivers (10/14) expressed various concerns regarding the use of the ML model to inform surgical decisions. The primary concern among caregivers (6/14) related to the current experimental stage of the model’s development; these caregivers stated they would feel more comfortable with model use if it were more established in clinical practice.

*“If someone said, ‘Oh, this is the first patient we’ve ever done this on. It’s experimental, and you’re patient number one,’ I might be a little more like, ‘Oh, okay.’…I would be comfortable with it…if it was made clear to me this is experimental, and then even more comfortable if it was like, ‘Okay, we are now in the phase where it’s standard practice.’”* Caregiver 13

Throughout interviews, caregivers consistently emphasized the substantial amount of trust they place in neurosurgeons to provide the best possible care for their loved one. A few caregivers (3/14) were concerned that neurosurgeons may rely too heavily on the model to inform surgical decisions, neglecting their own clinical judgment or consultation from colleagues. Caregivers reported that they would lose trust in their neurosurgeon’s expertise if they thought the neurosurgeon was over-relying on the model when making surgical decisions. Similarly, two caregivers doubted that the model could match the accuracy of the neurosurgeon’s clinical assessment, reiterating their preference for neurosurgeon expertise being applied alongside the model when making surgical decisions.

*“I would hope that he [the neurosurgeon] would also take the advice of AI but play it into his gut as well. Does he also agree with what the AI is saying? In any situation…I would hope that every person out there that uses AI goes in with a grain of caution and uses their own knowledge and their own gut feelings as to whether or not to trust it or to believe it or to use it.”* Caregiver 3

“*I'm gonna follow what my doctor experience is gonna show. Dr. [Neurosurgeon 1] is just amazing. He told us from day one what he thought, and he was right on the money, except for he was actually able to get 10 more percent than what he thought. That right there is—an AI can't do that. That is just a very talented surgeon who has been doing it for multiple years and have hands on [experience]…An AI cannot do that. I'm sorry. Well, AIs can do anything, obviously. For me personally, that is not what I would trust an AI over top of my surgeon*.” Caregiver 14

#### 3.3.3 Neurosurgeons

##### 3.3.3.1 Perceived benefits

Just under half of neurosurgeons (7/15) expressed at least one potential benefit to using the ML model to inform surgical decisions. Neurosurgeons felt that the model could help guide clinical decision-making (3/15) by providing additional information when making surgical decisions (2/15). Accurate estimates of patient outcomes for various surgical approaches, or opting for no surgery, were considered especially helpful in challenging cases, as neurosurgeons could use this additional data to better inform decision-making.

*“I think actually having more data is a good thing so this would be just another data point. I think at the end of the day, we would need to use all the information we have and then come up with our own conclusions as to what to do with that information. Obviously the better the information, the more likely I think neurosurgeons would use it*.” Neurosurgeon 8

Similarly, some neurosurgeons (3/15) felt that the additional information provided by the model could facilitate better conversations with patients and families who often have many questions about the risks and benefits of different surgical or treatment approaches. Neurosurgeons felt that the information provided by the model may help surgeons answer these questions more thoroughly and completely. One neurosurgeon felt the model may help patients better understand what their diagnosis means. Another agreed that a better understanding of these risks and benefits would help patients make informed decisions about their care.

“*Normally, especially for glioblastoma, patient and the family decide what to do. If we have all the info, more…well summarized info to provide them that will be super helpful. Because normally they ask a bunch of questions in the clinic…They all ask about the statistics. What's the risk of this surgery. What happens and honestly, it's all paper they report a different number and for me I would, it's a bit tough that I cannot give any definitive answer for that. It's quite ambiguous. I have to just give some info, but it's not really strictly giving what they really want. For example, if I can say, you know what, for this specific tumor in your age, it could be varied based on the pathology, but if you do surgery, this is the range of survival. The risk of having this amount of functional deficit is this percent. If I can get all the details and let them choose, then I think patient will be happy. I also will be very happy.”* Neurosurgeon 9

##### 3.3.3.2 Perceived concerns

Neurosurgeons (8/13) also expressed some concerns with using the ML model to inform surgical decisions. Similar to concerns with using the model to predict patient prognosis, neurosurgeons (4/15) were primarily concerned with the diversity of data sources and accuracy of data included in model development and training, including how inadequate data could lead to suboptimal GBM treatment.

These neurosurgeons emphasized the heterogeneity of outcomes among GBM patients, as well as the variability in treatment approaches from one hospital to another. Neurosurgeons felt the model’s training data should include a range of tumor types and surgical approaches from various hospitals to ensure the model can produce appropriate individualized recommendation plans. One neurosurgeon contemplated the impact that using the model to inform surgical decisions could have on patients and families. Specifically, they noted the model may categorize certain patients as inoperable, causing patients and their families to lose hope for potential positive outcomes not yet realized.

“*…there's so much nuance that I don't think that until we accrue more patients that we'll really be able to fully encompass the complexity of what a single patient looks like*.” Neurosurgeon 3

*“I also think that somebody can come in who statics [sic; statistics] say should go one way. I had a [patient] with a left thalamic glioblastoma. Had all the bad markers, all this kind of stuff…I felt like it was amenable to surgical resection. Most places would not resect this. He was a young guy. He wanted to live. I did it. I took it out. Case went great. Post-op imaging looked great. He had the worst markers. He was IDH wild-type. He was MGMT unmethylated. He had TERT promoter mutations. He lived over two years which is well beyond the average survivor for those things. My concern is that it puts you in a box. If you run his case through an AI algorithm, chances are he’d be predicted at three to eight months survival. He would not have to go surgical resection. That’s where I worry about things being becoming a self-fulfilling prophecy.”* Neurosurgeon 10

Some neurosurgeons (3/15) also expressed caution about relying too much on the model itself, rather than using it as a supplementary tool to their clinical judgment when making surgical decisions. While over-reliance on the model could cause neurosurgeons to miss important information or errors in model output, two neurosurgeons pointed out that neurosurgeons may not put much stock in the model to begin with. These individuals noted that neurosurgeons already have their own experience and guidelines that they trust to inform surgical decisions, which may make them less inclined to use the model or more likely to disregard its output. One neurosurgeon shared that their concerns about using the model for surgical decision-making were similar to those for predicting patient prognosis; they needed to understand the reasoning behind the model’s recommendations and how it arrived at certain conclusions.

*“I think it's tied to prognosis really. Ultimately in some ways the decisions are one and the same. If a patient has an exceedingly poor prognosis, then that will move us away from surgery because that may rob the patient of quality time. I think the two are sort of inextricable and I think as a neurosurgeon I would understand that. If the algorithm says the prognosis is poor, therefore surgery is not recommended, this would be consistent with my thought process anyway…I think that if there is discord between what the algorithm is recommending and what I would like to do, I would want to understand why is the algorithm recommending what it's recommending, particularly if the algorithm is recommending that I be more aggressive because aggression is associated with risk in our field.”* Neurosurgeon 5

##### 3.3.3.3 Unique perceptions

Two neurosurgeons did not express any particular benefit or concern about using the ML model to inform surgical decisions because they felt that using an algorithm was no different than other computer programs routinely used in their practice.

*“What’s new? We use computer. When you open your computer and you look at MRI, you’re using a computer algorithm to inform your decision because the MRI picture that you see is created on the loss of energy of electrons that have been affected by a magnetic field. That image doesn’t really exist. It’s generated by a computer. That’s using a computer algorithm to inform your decisions.”* Neurosurgeon 7

## 4 Discussion

This study engaged patients, caregivers, and neurosurgeons to understand their perceived benefits and concerns with using an ML model to predict GBM patient prognosis and inform surgical decisions. Triangulating the perspectives of these stakeholder groups is critical for ensuring that this new technology aligns with clinical realities while meeting the needs of patients and families (19). A multi-stakeholder approach is crucial for minimizing implementation risks and gaining a comprehensive understanding of challenges related to trust, acceptance, and integration of ML models into GBM clinical workflows.

Our study observed several areas of overlap in the perceived benefits and concerns for prognosis prediction and surgical decision-making across all three stakeholder groups. However, there were also differences in views within and between each group, highlighting the non-homogeneous nature of stakeholder perceptions. In what follows, we begin by discussing areas of agreement among stakeholders, explore differences within and between groups, and discuss the implications of our findings for future ML model development and implementation in GBM patient care.

Findings from these interviews suggest that using an ML model to predict prognosis for GBM patients can help neurosurgeons, patients, and families make informed treatment decisions. The model’s ability to quickly draw on large volumes of data to predict outcomes for individual patients provides faster, more comprehensive data processing than human assessment alone (6, 7). Participants across groups felt that, when used in conjunction with a neurosurgeon’s expertise, the ML model shows promise in helping neurosurgeons anticipate risks, answer patient questions, and deliver more precise treatment recommendations. Similarly, patients and caregivers felt that model-generated prognostic information could improve their understanding of the patient’s predicted quality of life and the various treatment pathways available to them—however these themes were less commonly mentioned among neurosurgeons. Future research would be beneficial to monitor the impact of ML-generated prognostic information on patient and caregiver knowledge and comprehension of this information.

The model shows particular benefit in helping patients weigh the risks and benefits of various surgical options against their own priorities. Informed decision-making supported by the model could lead to a better quality of life for patients if they and their loved ones are able to anticipate physical or cognitive needs over time (33). More robust prognostic information can facilitate better communication between doctors, patients, and families, supporting shared decision-making and greater patient satisfaction with their care (33). For patients and families, these benefits could lead to a greater sense of control in an otherwise uncontrollable, high-stakes, and emotional period of their lives.

However, all groups expressed concern over potential inaccuracies in the ML model’s predictions and the consequences these errors could have on decision-making and patient outcomes. Undetected errors or biases underlying the data used to generate the model’s predictions risk pursuing suboptimal treatment approaches or foregoing surgery with patients who would have otherwise benefited. Given the heterogeneity of individual GBM cases, this concern suggests that those implementing ML models in clinical settings should ensure that the model is trained on data that capture this heterogeneity, including diversity in patient demographics, tumor characteristics, and clinical presentations (34). Doing so can enhance the accuracy of model output and perceived usefulness to the user, improving user trust in the model for making important clinical decisions (34–37).

All groups, particularly neurosurgeons, acknowledged the risk of surgeons becoming over-reliant on the model rather than using it as a supplementary tool to predict prognosis or make surgical decisions. Patients and caregivers expressed substantial trust in their neurosurgeon to provide reliable recommendations and make clinical decisions. While some patients and caregivers felt that complete dependence on the model was unlikely or uncharacteristic of their neurosurgeon, they felt surgeons’ over-reliance on the model could erode trust. This concern underscores the need for neurosurgeons to provide clear, accurate explanations of how they use the ML model to interpret patient prognosis and make surgical decisions.

Relying solely on an ML model for prognostic or surgical decision-making, especially one that is experimental, is an understandably frightening prospect for patients. The risks to patient wellbeing that could arise from unchecked inaccuracies in the model are indeed too great for a model to replace human expert judgment. However, introducing an ML model into GBM surgical decision-making introduces a reciprocal risk of neurosurgeons disregarding the model’s accurate outcome predictions, raising questions about the appropriate level of reliance on the model for surgical decision-making. Neurosurgeons using this ML model will be tasked with finding a balance that grants credibility to the model output without it replacing expert clinical judgment.

Combining model use with strategies such as tumor boards or case conferences with other healthcare team members may help neurosurgeons establish a clear index of options and considerations, framing AI as a “thought partner” rather than a decision-maker (35, 38). For developers, employing frameworks such as the Artificial Intelligence Trust Framework and Maturity Model (AI-TMM) can help guide the design of transparent ML models, producing models that explain how they arrived at specific conclusions, as well as the confidence and patient-specific relevance of these conclusions (34, 39). These metrics can help healthcare providers detect errors and make informed decisions about whether to proceed with the model’s predictions (34, 39, 40). Future research should explore the enforcement of this balance in clinical settings, which likely has policy and legal implications regarding what parties (e.g., surgeons, hospitals, model developers) ought to be held accountable for clinical decisions when a ML model is involved (34, 41, 42).

Findings from this study also indicate that trust in and perceptions of the ML model will vary across individuals. For example, a few patients and caregivers felt that the model’s lack of emotion better facilitates unbiased information delivery. In contrast, others felt the lack of emotion makes the delivery of model output information feel cold, lacking a human component, during a very difficult and emotional conversation. A subset of patients and caregivers questioned whether they wanted to know prognostic information at all (33). For some, faith beliefs guided their perception of prognosis and their sense of hope for the future more than prognostic information generated by the model or neurosurgeon. This suggests that some patients and caregivers will not believe or want to receive prognostic information regardless of who generates it or how it is communicated. A patient’s personal information preferences are important for neurosurgeons to consider when determining how and whether to deliver ML-informed prognostic information. That is, neurosurgeons should ask patients about their preferences rather than assume what information they want regarding ML-informed prognosis. Future research is needed to understand which variables influence an individual’s preferences and the level of trust they place in ML models used for prognostic prediction.

Some participant concerns with the accuracy and applicability of the model were due to the early stage of development for this particular ML model. As a few neurosurgeons noted, the full impact of the model will remain unclear until it becomes more widely established in clinical practice. This suggests that concerns over accuracy and applicability of the model may subside over time as greater trust in the model is established. However, longitudinal research is needed to better understand the model’s performance and impact over time.

Some patients and caregivers expressed skepticism of AI in general, which may exacerbate mistrust of ML models used in clinical settings. These findings also imply that early-stage ML technologies face different challenges than their well-established counterparts. Trust in AI-driven clinical decision support develops through accumulated user experience and familiarity, peer endorsement and validation, and consistently accurate outputs over time – pathways that novel systems cannot offer at first launch (43). Early adoption of novel ML models requires special attention, and future research is needed to identify strategies for ML model developers and healthcare professionals to effectively communicate about model use to patients and caregivers.

This study also revealed several themes that were unique to the ML model’s ability to predict patient prognosis. More so than neurosurgeons, caregivers and patients discussed the important role that hope played in their coping and motivation throughout treatment and expressed concern that ML-informed prognostic information could cause some patients to lose that hope. Some also expressed concern that the model’s prognosis prediction may bias patient perceptions of their treatment plan; overly optimistic perceptions could cause patients to neglect certain realities about their situation, while overly negative perceptions might cause patients to become disengaged with treatment.

The timeline from GBM diagnosis to surgery is often brief, where patients and caregivers are tasked with processing complex and emotionally challenging information very quickly (44). During this short and difficult time, using or explaining the ML model could add to patients’ and families’ stress, uncertainty, and confusion rather than alleviate it. Neurosurgeons and other healthcare professionals who facilitate prognostic conversations must consider how this information is conveyed to and interpreted by the patient. This finding emphasizes the importance of the doctor-patient relationship and maintaining the “human touch” in clinical care when an ML model is involved. Communicating the model’s accuracy and limitations to patients and caregivers in accessible terms can help contextualize the model’s predictions for each patient.

Further, only one patient shared a concern with privacy and the use of patient data by the ML model. Because GBM is a terminal illness with few treatment options, patients who wish to capitalize on the benefits of the model are placed in a vulnerable position to agree to the terms of their data use, potentially without understanding or agreeing with the potential uses of their data. In prior research, data privacy and use has been a low or nonexistent priority for patients facing terminal diagnoses, which may explain why this theme was rare (45, 46). However, data privacy and protection of sensitive data remains a key factor influencing public trust in ML systems (39). Healthcare institutions and developers of ML models still bear responsibility for the stewardship and appropriate use of patient data from ML models and must ensure that patient data privacy and protection from harmful use (e.g., insurance coverage decisions) is upheld long-term.

A few neurosurgeons thought this ML model is no different from any other software or tool used in clinical practice, and the AI component of the model does not require special consideration. This view may hold some validity – for instance, past research has observed challenges related to communication between neurosurgeons and patients regarding prognosis discussions, regardless of the involvement of AI tools (47). However, it is essential to recognize the unique aspects of introducing ML models into GBM prognosis and decision-making. Along with prior literature, this study highlighted the potential risks of over-reliance on machine learning systems for prognostication and surgical decision-making, emphasizing the need for careful consideration of how these models are implemented and communicated (48). Indifference to the model’s unique attributes could lead to unintended consequences, such as underplaying model biases or exacerbating communication gaps. Successful integration of ML models into clinical practice requires thoughtful consideration of its effects, both positive and negative, on clinical operations and patient outcomes. Model developers and neurosurgeons alike will be tasked with ensuring that beneficiaries of the model are equipped with the knowledge and skills required to capitalize on its benefits without exacerbating risks to clinical operations or patient wellbeing.

### 4.1 Limitations

This study has several limitations to consider when interpreting interview findings. The qualitative nature and sample size of this study prohibit statistical comparisons, thereby limiting generalizability. While our sample represented a variety of patient ages, there may be differing preferences and perceptions among those of different races, ethnicities, or other individual differences, further limiting generalizability of our findings. Additionally, patients and caregivers were primarily recruited after receiving treatment from a single clinical location, which may limit the applicability of findings to other geographic regions or clinical settings. All patients and caregivers in this study reported having strong social support systems, and there may be differing perceptions among those without such support.

Neurosurgeons in this sample were predominantly male (87%) and identified as White (53%) or Asian (47%). While this demographic distribution is consistent with the gender and racial representation among neurosurgeon residency programs in the US, it precludes the comparison of neurosurgeon viewpoints across gender and race (49).

Participants mentioned overall fewer benefits and concerns with using an ML model to predict surgical outcomes compared to prognosis prediction. This difference may be due to the arrangement of the interview guide in which prognosis questions were asked first. However additional research is needed to determine whether other factors, such as the emotional impact of prognostic information versus the safety risks of surgical resection, affect this difference. Further, the way in which the ML model was presented at the beginning of interviews, including information about high model accuracy, may have influenced participant responses. Future research should explore how ML model accuracy affects perceptions of ML models.

### 4.2 Conclusion

This study demonstrates the importance and value of multi-stakeholder engagement in the development of ML models used to support clinical decision-making. We found that for both GBM prognostication and surgical decision-making, patients, caregivers, and neurosurgeons appreciated the benefit of having an ML model available to synthesize vast quantities of patient data to generate precise information to aid in shared decision-making for individual patients. However, the consequences of biases in model training data, inaccurate model predictions, or inappropriate reliance on model predictions have significant implications for patient outcomes. Thus, ML models cannot be the only tool used for prognosis prediction or surgical decision-making. It is prudent for neurosurgeons to use their own professional judgment to interpret model output in the context of each unique patient and communicate with patients in a nuanced and effective way about the implications of model output.

Further, prognostic conversations in GBM cases are difficult and deeply emotional for patients and their families, which can have a serious impact on a patient’s sense of hope and ability to process complex information. Regardless of whether an ML model is involved, neurosurgeons must approach these conversations with empathy, taking time to help patients understand what their prognosis might mean for them. However, the introduction of an ML model warrants additional discussion of how neurosurgeons use the model in conjunction with their own clinical judgment in order to maintain trust within the doctor-patient relationship. Developers of ML models and the neurosurgeons who use them have a responsibility to ensure that models are transparent, accurate, and unbiased in their calculations and output – particularly during the early stages of development and integration. This commitment will support responsible and successful development and implementation of ML models in clinical decision-making.

## Data Availability

The datasets presented in this study can be found in online repositories. The names of the repository/repositories and accession number(s) can be found at: https://doi.org/10.5064/F60VEORO.
